# The Interaction of Insulin and Pituitary Hormone Syndromes

**DOI:** 10.3389/fendo.2021.626427

**Published:** 2021-04-28

**Authors:** Marie Helene Schernthaner-Reiter, Peter Wolf, Greisa Vila, Anton Luger

**Affiliations:** Clinical Division of Endocrinology and Metabolism, Department of Medicine III, Medical University of Vienna, Vienna, Austria

**Keywords:** insulin, cortisol, growth hormone, prolactin, thyroid hormones, sex hormones

## Abstract

Pituitary hormone axes modulate glucose metabolism and exert direct or indirect effects on insulin secretion and function. Cortisol and growth hormone are potent insulin-antagonistic hormones. Therefore impaired glucose tolerance, elevated fasting glucose concentrations and diabetes mellitus are frequent in Cushing’s disease and acromegaly. Also prolactinomas, growth hormone (GH) deficiency, hypogonadism and hypothyroidism might be associated with impaired glucose homeostasis but usually to a lesser extent. Therefore glucose metabolism needs to be closely monitored and treated in patients with pituitary adenomas. Correction of the pituitary dysfunction is frequently followed by improvement of glucose homeostasis.

## Introduction

Pituitary hormone excess and deficiency syndromes impair the action of insulin to a varying extent by direct and indirect effects. This is most prevalent and most pronounced in Cushing’s disease and acromegaly but also observed in prolactinomas, GH-deficiency, hypogonadism and hypothyroidism (1). The impairment of glucose metabolism is related to the extent and duration of hormone excess and contributes to the co-morbidities, increased cardiovascular risk and to the increased mortality in patients with pituitary adenomas (2, 3). Occasionally impaired glucose tolerance or diabetes mellitus is the presenting symptom of Cushing’s syndrome or acromegaly (4, 5). In many instances, cure or amelioration of the hormonal imbalance by pituitary surgery, medical or radio-therapy induces improvement of glucose homeostasis (6, 7). On the contrary, some drugs used for therapy of pituitary adenomas or hormone deficiencies negatively impact glucose metabolism (8). In any case parameters of glucose metabolism should be closely monitored at presentation and throughout active disease but also after cure of the underlying pituitary disease. Consequent treatment of diabetes mellitus, impaired glucose tolerance and elevated fasting glucose concentrations is mandatory and in the absence of strong evidence for these special conditions should in general follow established guidelines for the general population. This article summarizes the effects of functioning and non-functioning pituitary adenomas on glucose metabolism.

## Cushing’s Disease

**Figure 1:**
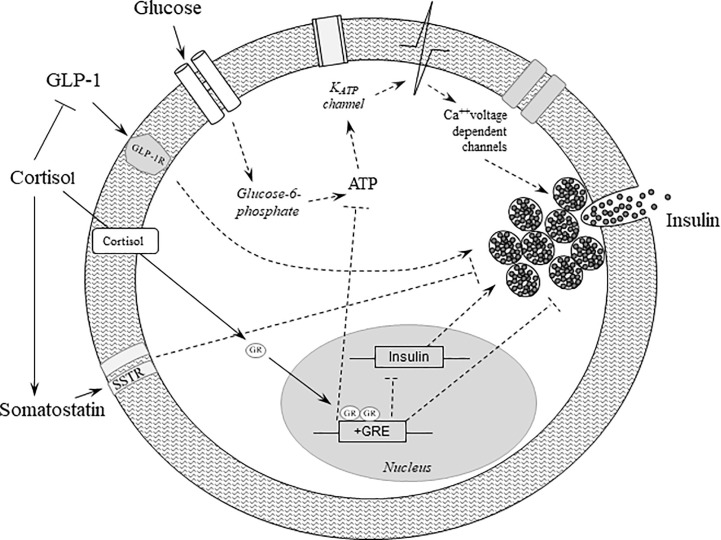
Effects of glucocorticoids on beta-cell function. Binding of glucose to the GLUT2 receptor increases oxidative metabolism and ATP synthesis, thereby leading to the closure of the K_ATP_ channels, which in turn stimulates membrane depolarization and insulin secretion. Cortisol binds to and activates the glucocorticoid receptor, which translocates to the nucleus and initiates several cascades culminating in repression of insulin transcription and inhibition of insulin release. In addition, cortisol further deteriorates beta-cell function by reducing GLP-1 production and its positive effects on insulin secretion, and by increasing the secretion of somatostatin, which in turn negatively impacts insulin gene transcription and insulin secretion. Dashed lines represent indirect effects. ATP, adenosine triphosphate; Ca, Calcium; GLP-1, glucagon-like peptide 1; GLP-1R, glucagon-like peptide 1 receptor; GLUT2, glucose transporter 2; GR, glucocorticoid receptor; GRE, glucocorticoid response element.

Interactions between insulin and cortisol are manifold (2). Cortisol is a potent insulin-antagonistic hormone inhibiting insulin secretion, stimulating glucagon secretion and disrupting insulin signaling. Cortisol inhibits insulin release and reduces GLP-1 production and thereby also insulin secretion (**Figure 1**). Cortisol induces the expression of key gluconeogenic enzymes and increases hepatic glucose production and gycogenolysis (**Table 1**). In the muscle cortisol reduces the translocation of the insulin-dependent glucose transporter 4 (GLUT4) to the plasma membrane thereby impairing glucose uptake and by activating glycogen synthase kinase-3 suppresses glycogen synthesis and promotes protein degradation (9) (**Table 1**). In addition cortisol’s lipolytic activity with increase in circulating free fatty acids contributes to insulin resistance (**Table 1**). Thus it is not surprising that cortisol excess is associated with disturbed glucose homeostasis in patients with Cushing’s disease. Impaired glucose tolerance has been reported in 21-64% at diagnosis and diabetes mellitus in 20-47% (10–15). No data are available in the literature on the percentage of patients requiring insulin therapy at diagnosis. But there are several reports on diabetic ketoacidosis as a presenting symptom of Cushing’s disease where diabetes was cured or remitted with the control of cortisol excess (4, 16, 17). Possible explanations are cortisol’s stimulation of lipolysis and increase in free fatty acids, suppression of insulin secretion and ketogenesis in the liver.

**Table 1:** Metabolic effects of insulin and alterations in Cushing‘s disease and acromegaly.

Organ	Insulin	Cushing‘s disease	Acromegaly
**Liver**			
Gluconeogenesis Glycogenolysis	↓ ↓	↑ ↑	↑ ↑
**Muscle**			
Glucose uptake Proteolysis Glycogen synthesis	↑ ↓ ↑	↓ ↑ ↓	↓ ↓ ↑
**Adipose Tissue**			
Lipolysis Glucose uptake	↓ ↑	↑ ↓	↑ ↓

Vice versa, in a series of 200 overweight patients with type 2 diabetes and a HbA1c >8% devoid of specific clinical symptoms of Cushing’s syndrome, 17 patients were identified with 2 abnormal screening tests for hypercortisolism but only 3 patients received a definitive diagnosis of Cushing’s disease while 1 patient had a cortisol producing adrenal adenoma (18). In addition, a recent study in 384 newly diagnosed patients with type 2 diabetes without a persuasive cushingoid phenotype reported a 5% prevalence of hypercortisolism including 1 patient with a pituitary macroadenoma and 9 patients with an adrenal adenoma (19). The low prevalence of Cushing’s disease in patients with type 2 diabetes and the high prevalence of type 2 diabetes in the general population, does not justify the recommendation that all type 2 diabetes patients should be screened for hypercortisolism, but it should be borne in mind even in the absence of characteristic signs, symptoms and comorbidities. An increased activity of the hypothalamic-pituitary adrenal axis has been observed in patients with type 2 diabetes mellitus with asymptomatic autonomic imbalance with prevalent parasympathetic failure (20). Hypercortisolism may also be caused by alcoholism, severe depression, anxiety disorders and severe obesity (21).

Remission or reduction of hypercortisolism by transsphenoidal surgery, medical - or radio-therapy frequently improves glucose homeostasis but induces remission of diabetes or prediabetes in only a minority of patients (6, 7, 22, 23). This might be related to the delay of diagnosis and thus prolonged exposure to cortisol excess especially in patients predisposed to diabetes mellitus. In contrast, the second generation somatostatin analog pasireotide has substantial negative effects on glucose homeostasis despite a reduction or normalization of hypercortisolism (8, 24). Pasireotide’s higher affinity to somatostatin receptor subtype 5 (SSTR5) and lower affinity to SSTR2 compared to first generation somatostatin analogs is presumed to result in a greater suppression of insulin secretion and lesser decrease in glucagon secretion (25). In addition pasireotide suppresses also the secretion of GLP-1 and GIP (26). Close monitoring of glucose concentrations after initiation and throughout therapy with pasireotide treatment is mandatory (8, 24). Mifepristone, a glucocorticoid and progesterone receptor antagonist, has been shown to improve insulin sensitivity and decrease fasting plasma glucose concentrations and HbA1c concentrations in patients with Cushing’s disease and impaired glucose tolerance or diabetes mellitus (27).

No strong evidence on how to treat disturbed glucose metabolism in Cushing’s disease exists. Considering pathophysiological mechanisms insulin sensitizing drugs counteracting insulin resistance should be considered as first choice. This has also been suggested by a guideline of the Italian Society for the Study of Diabetes and the Italian Endocrinological Society (28). Due to the negative effects of thiazolidinediones on bones (29) and fluid retention which would add to the ones of hypercortisolism metformin either as monotherapy or, if glycemic targets cannot be reached, in combination with other oral antidiabetic drugs, GLP-1 receptor agonists or insulin should be considered as first choice. SGLT-2 inhibitors might induce the so called “euglycemic” ketoacidosis and therefore appear not to be optimally suited. They add to cortisol’s metabolic actions by increasing glucagon secretion and ketogenesis in the liver as well as reabsorption of ketone bodies in the kidney (30) and their side effect of genitourinary infections may be especially disadvantageous in patients with Cushing’s syndrome who are already at an increased risk of infection. In contrast, GLP-1 receptors agonists which besides their insulin stimulating effect also suppress glucagon secretion and appetite appear to be an attractive alternative. GLP-1 infusion in a patient with Cushing’s disease induced diabetes has indeed demonstrated similar insulinotropic, glucagonostatic and glucose-lowering actions as in patients with type 2 diabetes (31).

## Acromegaly

Growth hormone (GH) induces lipolysis with increased circulating free fatty acids leading to insulin resistance in the liver and peripheral muscle (32–34) (**Table 1**). Thereby hepatic glucose production, gluconeogenesis and glycogenolysis in the liver are stimulated, whereas glucose uptake in the muscle is reduced (**Table 1**). The contribution of GH-induced elevated IGF-1 concentrations to these effects is not completely elucidated (34). While IGF-1 has insulin-agonistic actions and has been used for treatment of severe insulin resistance, IGF-1 reduction with the GH antagonist pegvisomant improves glycemic control in acromegalic patients with diabetes or impaired glucose tolerance despite elevated GH (35, 36).

GH excess leads to impaired glucose homeostasis in a considerable part of patients with acromegaly. In more than half of the patients, diabetes is diagnosed before acromegaly (37) highlighting the need to increase the awareness of this association. At diagnosis, 22 – 40.5% of patients with acromegaly exhibit elevated fasting glucose concentrations and/or impaired glucose tolerance and 12 – 34.9% diabetes mellitus (37–42). In two studies that reported details on diabetes treatment the percentage of patients requiring insulin therapy ranged from 17.6 to 24% (37, 41). Also several cases of diabetic ketoacidosis have been reported as presenting symptom/condition in patients with acromegaly (5, 43–46). This association must be attributed to extremely severe insulin resistance as well as GH’s lipolytic action with an increase in free fatty acids and the suppression of insulin secretion. In all reported cases of acromegaly-associated ketoacidosis, diabetes resolved after remission of acromegaly.

Glucose homeostasis frequently improves following remission of GH excess by transsphenoidal surgery or pharmacological disease control but glucose metabolism remains impaired in many patients (47, 48).

Pharmacological treatment of acromegaly has varying effects on glucose homeostasis. Treatment of mild acromegaly with bromocriptine has been reported to be associated with favorable effects that are more pronounced with the more potent dopamine agonist cabergoline (49, 50). Most studies evaluating the first generation somatostatin analogs have demonstrated neutral to mild adverse effects (51). The second generation somatostatin analog pasireotide has been shown to induce impaired glucose tolerance, overt diabetes or worsen glycemic control especially in patients with pre-existing disturbed glucose metabolism (52). The mechanisms by which pasireotide exerts these effect have been described in the section on Cushing’s disease. In contrast, the GH-antagonist pegvisomant has been shown to improve glucose metabolism whether applied as monotherapy or added to treatment with somatostatin analogs (36).

As with Cushing’s disease, no clear evidence exists as to the optimal treatment of impaired glucose tolerance and diabetes associated with acromegaly. From a pathophysiological viewpoint substances counteracting insulin resistance appear to be the first choice also in acromegaly (53). Metformin should be preferred to glitazones due to the adverse effects on bone and fluid retention of the latter. As mentioned in the section on Cushing’s disease this has also been supported by guideline of the Italian Society for the Study of Diabetes and the Italian Endocrinological Society (28). SGLT2 inhibitors appear not to be the first choice due to their mechanism of action adding to GH’s effects on glucose metabolism by facilitating the so-called euglycemic ketoacidosis (see Cushing section). In fact, a case of rapid onset diabetic ketoacidosis has been reported after addition of the SGLT2 inhibitor empaglflozin to metformin, sitagliptin and gliclazide in a patient with unrecognized acromegaly and type 2 diabetes (54). DPP4-inhibitors and GLP-1 receptor agonists might be used along with metformin but insulin remains the therapy of choice in cases with severe hyperglycemia.

## Effects of Excess/Deficiency of Other Pituitary Hormones on Glucose Homeostasis

In contrast to the frequent and sometimes severe effects of hypercortisolism and GH excess, the deficiency or excess of other pituitary hormones have also been shown to affect glucose homeostasis but mostly to a less severe extent. In these hormonal imbalances factors regulating insulin sensitivity and secretion are often acting in opposite directions and affected by concomitant deficiencies of other pituitary hormones. In addition to the extent of hormone deficiency or excess, changes in appetite regulation, energy expenditure, body weight and body composition might contribute to the adverse effect on glucose homeostasis. It is therefore not possible to reduce observed clinical changes to single pathomechanisms that are often derived from animal or *in vitro* studies and might not apply to humans. In the following part some of the postulated pathways that might contribute to the impaired glucose homeostasis will be briefly discussed.

Whereas GH deficiency according to the negative effects of elevated GH levels on glucose homeostasis would be expected to result in beneficial effects the findings of available studies on this topic are inconsistent. Normal, reduced as well as increased insulin sensitivity have been reported in GH deficient patients (55, 56). GH deficiency in adults has been reported to induce insulin resistance by inhibiting glucose storage rate and glycogen synthase activity in peripheral tissues (57). In addition, increased fat mass and decreased muscle mass contribute to insulin resistance in these patients. In a retrospective analysis of 6050 patients with adult-onset GH deficiency, a significantly increased prevalence of diabetes has been reported (58).

Hyperprolactinemia is associated with insulin resistance which improves with dopamine agonist therapy which might be related to the therapy associated weight loss and an activation of insulin signaling (59–62). The pathomechanisms by which prolactin impairs glucose homeostasis are unclear, some effects would rather enhance insulin sensitivity. Central prolactin infusion in rats increases food intake, but prolactin receptor knockout mice show reduced adiposity and prolactin deficiency had only negligible effects on body weight, body composition, serum lipids and adiponectin concentrations (63, 64). It also has been demonstrated that prolactin induces increased expression of *Pparg*, *Xbp1s*, and *GLUT4* in visceral and subcutaneous adipose tissue and elevated circulating adiponectin levels thereby increasing insulin sensitivity in mice (65). Prolactin induced hypogonadism might contribute to the metabolic alterations in hyperprolactinemia.

Gonadotrophin deficiency due to mass effects of pituitary adenomas or gonadotrophin suppression due to cortisol or prolactin excess might induce insulin resistance in men and women that can be improved by hormone replacement therapy. This can be deduced from studies in men and women with hypogonadism unrelated to pituitary diseases (66–69). Pathophysiological mechanisms by which testosterone deficiency might impair glucose metabolism in men include reduced insulin receptor expression and intracellular insulin signaling, reduced GLUT4 expression and membrane translocation in skeletal muscle and liver cells as well as in adipose tissue (70, 71). Also key enzymes of the glycolysis pathway and mitochondrial oxidative phosphorylation are increased by testosterone. In addition, promotion of differentiation of pluripotent stem cells into adipocytes rather than myocytes in testosterone deficiency and the resulting increase in fat mass and decrease in muscle mass might contribute to development of insulin resistance (71). Estrogen deficiency might impair glucose homeostasis through several pathomechanisms. 17β estradiol has been shown to suppress hepatic glucose production and gluconeogenesis through inhibition of the transcription factor Foxo1via activation of estrogen receptorα-phosphoinositide 3-kinase-Akt signaling (72). Estradiol improves also insulin-induced glucose uptake and GLUT4 translocation to the plasma membrane in adipocytes (73). In addition estrogens are also involved in the regulation of energy expenditure and food intake and have been reported to protect pancreatic β cells from various injuries as well as enhancing insulin biosynthesis through activation of the estrogen receptorα (74).

Hypothyroidism has also been associated with insulin resistance and risk of type 2 diabetes. Insulin resistance could be restored by thyroid hormone replacement (75, 76). The interplay between thyroid hormones and appetite regulation, energy expenditure, sympathetic activity and glucose and lipid metabolism is complex (77). Among other effects T3 exerts an acute hypoglycemic effect by activating the phosphatidylinositol 3-kinase signaling cascade which is also involved in lowering serum and hepatic triglycerides. Thyroid hormones cause an increase in ATP utilization accelerating lipolysis/fatty acid oxidation and increased protein turnover. T3 increases also the translocation of GLUT4 to the plasma membrane in skeletal muscle and adipose tissue and this might contribute to the hypothyroidism associated impaired glucose tolerance (77).

In conclusion, disturbances of glucose metabolism are frequently found in patients with pituitary adenomas, and can be especially severe in Cushing’s disease and acromegaly. Hyperglycemia and diabetes have serious consequences increasing cardiovascular morbidity and mortality and therefore deserve special attention and consequent treatment at any stage of the disease. Cardiovascular endpoint studies have revolutionized the therapy of patients with diabetes, but they are lacking in the context of diabetes due to pituitary diseases. Therapy of the underlying pituitary disease frequently ameliorates glucose homeostasis.

## Author Contributions

All authors contributed to the article and approved the submitted version.

## Conflict of Interest

The authors declare the following conflicts of interest: MS-R has received honoraria for presentations from HRA Pharma; GV has received lecture and/or consulting fees from Ipsen, Pfizer, Novo Nordisk, Recordati, Takeda and HRA Pharma, and is Research Investigator in studies sponsored by Novartis, Recordati, Corcept, Chiasma and Takeda; AL has received honoraria lecture and/or consulting fees from Ionis, Ipsen, Merck, Novartis, Pfizer, Sandoz.

The remaining author declares that the research was conducted in the absence of any commercial or financial relationships that could be construed as a potential conflict of interest.
